# Heart Failure Management in 2023: A Pharmacotherapy- and Lifestyle-Focused Comparison of Current International Guidelines

**DOI:** 10.1016/j.cjco.2023.05.008

**Published:** 2023-05-26

**Authors:** Blair J. MacDonald, Sean A. Virani, Shelley Zieroth, Ricky Turgeon

**Affiliations:** a University of British Columbia, Vancouver, British Columbia, Canada; b University of Manitoba, Winnipeg, Manitoba, Canada

## Abstract

This review examines the pharmacotherapy and lifestyle recommendations of the most recent iterations of the Canadian Cardiovascular Society (CCS) / Canadian Heart Failure Society (CHFS), the European Society of Cardiology (ESC), and the American Heart Association (AHA) / American College of Cardiology (ACC) / Heart Failure Society of America (HFSA) heart failure (HF) guidelines, which all have been updated in response to therapeutic developments across the spectrum of left ventricular ejection fraction. Identified areas of unanimity across these guidelines include the following: recommending quadruple therapy for patients with HF with reduced ejection fraction (HFrEF; although no guideline proposed an ideal sequence of initiation); intravenous iron administration for patients with HFrEF and iron deficiency; and sodium restriction for patients with HF. Recent evidence regarding the harms of HFrEF medication withdrawal in patients with HF with improved ejection fraction has prompted subsequent guidelines to recommend against withdrawal. Due to the lower quality of evidence, there are disagreements regarding management of HF with preserved ejection fraction and uncertainty regarding management of HF with mildly reduced ejection fraction. Practical guidance is provided to clinicians navigating these challenging areas. In addition to these clinically focused comparisons, we describe opportunities for guideline improvement and harmonization. Specifically, these include opportunities regarding HFrEF sequencing, the need for timely updates, shared decision-making, Grading of Recommendations, Assessment, Development and Evaluations (GRADE) framework adoption, and the creation of recommendations where high-quality evidence is lacking. Although these guidelines have broad agreement, key areas of controversy remain that may be addressed by emerging evidence and changes in guideline methodology.

## Résumé

Le présent examen porte sur les recommandations en matière de pharmacothérapie et d’habitudes de vie en présence d’insuffisance cardiaque (IC) des plus récentes lignes directrices sur l'IC de la Société cardiovasculaire du Canada (SCC) / Société canadienne d’insuffisance cardiaque (SCIC), de la Société européenne de cardiologie (ESC) et de l’American Heart Association (AHA) / American College of Cardiology (ACC) / Heart Failure Society of America (HFSA), qui ont toutes été mises à jour en réponse aux avancées thérapeutiques pour tout le spectre de fractions d’éjection du ventricule gauche. Voici certains des domaines qui font l’unanimité dans ces lignes directrices : quadrithérapie recommandée chez les patients atteints d’IC avec fraction d’éjection réduite (ICFER; toutefois, aucune ligne directrice ne comporte une proposition concernant la séquence idéale de l’instauration du traitement); administration de fer par voie intraveineuse aux patients atteints d’ICFER présentant une carence en fer; et restriction de l’apport sodique chez les patients aux prises avec une insuffisance cardiaque. Les données probantes récemment publiées sur les effets nocifs de l’abandon de médicaments contre l’ICFER chez les patients atteints d’insuffisance cardiaque dont la fraction d’éjection s’est améliorée ont incité les organismes à déconseiller de tels abandons dans leurs recommandations subséquentes. En raison de la faible qualité des données, il existe des différends en ce qui a trait à la prise en charge de l’IC avec fraction d’éjection préservée, ainsi qu’une incertitude quant à la prise en charge de l’IC avec fraction d’éjection légèrement réduite. Des conseils pratiques sont fournis afin d’aider les cliniciens à gérer ces domaines complexes. En plus de ces comparaisons axées sur la pratique clinique, nous décrivons des occasions d’amélioration et d’harmonisation des lignes directrices. Plus précisément, ces occasions touchent la séquence des traitements de l’ICFER, le besoin de mises à jour opportunes, la prise de décision conjointe, l’adoption du cadre GRADE (*Grading of Recommendations, Assessment, Development and Evaluations*, soit la classification des recommandations, des appréciations, du développement et des évaluations), ainsi que l’élaboration de recommandations dans les domaines où des données probantes de haute qualité sont lacunaires. Bien que ces lignes directrices obtiennent un vaste accord, d’importants domaines controversés demeurent. La publication de données probantes et la modification des méthodes d’élaboration des recommandations pourraient cependant y remédier.

The management of heart failure (HF) is becoming increasingly complex. Over the past decade, major developments have broadened available treatment options across the spectrum of left ventricular ejection fraction (LVEF). This expansion includes advancements in the management of HF with reduced ejection fraction (HFrEF) and HF with preserved ejection fraction (HFpEF), and the more recent introduction of HF with mildly reduced ejection fraction (HFmrEF) and HF with improved ejection fraction (HFimpEF).^1^^,^^2^ Mirroring the rapid pace of these advances, international HF guidelines have recently undergone significant revisions to provide contemporary guidance on pharmacologic HF management.

The most recent comprehensive HF guideline document by the Canadian Cardiovascular Society (CCS) was published in 2017,^3^ with subsequent focused updates published in 2020 and 2021, with the 2021 update substantively changing the standard of care for HFrEF.^4^^,^^5^ The most recent comprehensive HF guideline documents from the European Society of Cardiology (ESC) and the American Heart Association (AHA)/American College of Cardiology (ACC)/Heart Failure Society of America (HFSA)—which supersede previous recommendations—were first published in 2021 and 2022, respectively.^6^^,^^7^ Although these international HF guidelines have broad concordance, they also have important differences in focus, scope, and methodology. Given this, a comparison of society recommendations and the underlying evidence can provide insights on international consensus, opportunities to support harmonization, and gaps in knowledge and guidance.

Here, we compare the pharmacotherapy and lifestyle recommendations of the latest CCS, ESC, and AHA/ACC/HFSA HF guidelines and discuss supporting evidence to inform clinicians seeking practical guidance amidst areas of uncertainty and disagreement. This review adds to an existing article that compared the ACC/AHA/HFSA and ESC heart failure guidelines,^8^ by adding comparisons to the CCS guidelines, offering a Canadian perspective on guidance and emerging evidence, and expanding the discussion on pharmacotherapy for HFpEF/HFmrEF. Additionally, we discuss areas for guideline improvement, including HFrEF sequencing, timely updates, shared decision-making, adoption of the Grading of Recommendations, Assessment, Development and Evaluations (GRADE) approach, and recommendations in areas without high-quality evidence. Table 1 provides a summary of takeaways for HF management and guideline development.

**Table 1:** Top 5 clinical and guideline development takeaways from recent heart failure guidelines

	Takeaways for heart failure management	Takeaways for guideline development
1.	• For HFrEF, there is universal agreement to initiate quadruple therapy as soon as possible, but the best sequencing strategy remains elusive.	• In the absence of comparative RCTs, model-based estimates of the benefits of different HFrEF sequencing strategies could inform future guideline recommendations.
2.	• For HFimpEF, the ESC and AHA/ACC/HFSA guidelines recommend against withdrawing HFrEF pharmacotherapy, based on trial results published after the 2017 CCS guidelines.	• Guideline recommendations and discussions may not incorporate the latest evidence on a topic (at least until a subsequent update). Systematic update efforts are necessary to ensure clinicians are informed as new evidence emerges.
3.	• For HFpEF, the certainty of evidence for pharmacotherapy is low at best (except for SGLT2i initiation), resulting in guideline discordance. • This is an opportunity for clinicians to engage patients with HFpEF in shared decision-making, using decision aids and patient-oriented outcome data.	• Recommendations should be accompanied by sufficient information on treatment benefits and harms to empower clinicians to engage patients in shared decision-making.
4.	• For HFmrEF, differences between guidelines are attributable to the rapid pace of evidence advancement (with the AHA/ACC/HFSA being the most up to date). • The ESC and AHA/ACC/HFSA make weak recommendations in favour of ARB/ARNI, beta-blockers, and MRAs. • The AHA/ACC/HFSA also makes a moderate-strength recommendation in favour of SGLT2i’s.	• The GRADE certainty-of-evidence rating system provides greater transparency and allows for more-nuanced interpretations than the traditional ACC/AHA levels of evidence.
5.	• There are unanimous recommendations for intravenous iron in the treatment of iron deficiency with HFrEF. • The clinical benefits of sodium restriction are unclear. In SODIUM-HF (published after all 3 guidelines),^9^ strict sodium restriction did not lead to fewer HF events, compared to usual care (∼2 g/d).	• Intravenous iron for patients with iron deficiency and HFrEF has the greatest impact on quality of life of any HFrEF pharmacotherapy, a priority for patients with HFrEF.^10^ Future guideline iterations should feature this therapy more prominently. • Incorporation of emerging evidence on the preferences and values of patients with HFrEF may further guide the prioritization of interventions. • In the absence of evidence comparing the efficacy of different sodium targets, nuanced weak recommendations based on reasonable ranges may still be possible (eg, sodium intake target of ∼1.4–3 g/d). When this is not feasible, it is valuable to strongly recommend shared decision-making informed by transparent disclosure of the uncertainty regarding potential benefits and harms.

ACC, American College of Cardiology; AHA, American Heart Association; ARB, angiotensin receptor blocker; ARNI, angiotensin receptor-neprilysin inhibitor; CCS, Canadian Cardiovascular Society; EF, ejection fraction; ESC, European Society of Cardiology; GRADE, Grading of Recommendations, Assessment, Development and Evaluations; HF, heart failure; HFimpEF, HF with improved EF; HFmrEF, HF with mildly reduced EF; HFpEF, HF with preserved EF; HFrEF, HF with reduced EF; HFSA, Heart Failure Society of America; MRA, mineralocorticoid receptor antagonist; RCT, randomized controlled trial; SGLT2i, sodium/glucose cotransporter-2 inhibitor; SODIUM-HF, **S**tudy **O**f **D**ietary **I**ntervention **U**nder 100 **m**mol in **H**eart **F**ailure trial.

## Assessment of Evidence and Strength of Recommendations

Societies differ in their guideline methodology for determining certainty (or “quality”) of evidence and the strength of recommendations (Table 2).^15^, ^16^, ^17^ The CCS uses GRADE, whereas the AHA/ACC/HFSA guidelines use the ACC/AHA classification, and the ESC uses a modified version of the ACC/AHA classification. These methodologies are conceptually similar; however, discordance occurs due to differences in category definitions (eg, what constitutes strong vs moderate certainty/quality of evidence) and application of judgement by guideline panels.

**Table 2:** Comparison of evidence rating systems used in heart failure guidelines

CCS/CHFS	ESC	AHA/ACC/HFSA
GRADE certainty of evidence: Rated as high, moderate, low, or very low.	Comparable classification using “level of evidence”	
**High**: Further research is very unlikely to change our confidence in the estimate of effect. No serious risk of bias, inconsistency, imprecision, indirectness, or publication bias	**A:** Data derived from multiple randomized clinical trials or meta-analyses	**A:** High-quality evidence from more than 1 RCT, meta-analyses of high-quality RCTs, or one or more RCTs corroborated by high-quality registry studies
**Moderate**: Further research is likely to have an important impact on our confidence in the estimate of effect and may change the estimate.	**B:** Data derived from a single randomized clinical trial or large nonrandomized studies	**B:** • **R:** Moderate-quality evidence from 1 or more RCTs, or meta-analyses of moderate-quality RCTs • **NR:** Moderate-quality evidence from 1 or more well-designed, well-executed nonrandomized studies, observational studies, or registry studies; meta-analyses of such studies
**Low**: Further research is very likely to have an important impact on our confidence in the estimate of effect and is likely to change the estimate.	-	**C-LD:** Randomized or nonrandomized observational or registry studies with limitations of design or execution; meta-analyses of such studies; physiological or mechanistic studies in human subjects
**Very low:** Any estimate of effect is very uncertain.	**C:** Consensus of opinion of the experts and/or small studies, retrospective studies, registries	**C-EO:** Consensus of expert opinion based on clinical experience
GRADE strength of recommendation: CCS uses strong or weak as qualifiers of strength of recommendations based on consideration of quality of evidence, difference between desirable and undesirable effects (ie, tradeoffs), values and preferences, and cost or value considerations.	Comparable classification of strength of recommendation	
Strong recommendation in favour of an intervention	**Class I:** Evidence and/or general agreement that a given treatment or procedure is beneficial, useful, effective	**Class 1 (strong):** Benefit > > > Risk
Weak recommendation in favour of an intervention	**Class II:** Conflicting evidence and/or a divergence of opinion about the usefulness/efficacy of the given treatment or procedure • **IIa:** Weight of evidence/opinion is in favour of usefulness/ efficacy • **IIb:** Usefulness/efficacy is less well established by evidence/opinion	**Class 2a (moderate):** Benefit > > Risk **Class 2b (weak):** Benefit ≥ Risk
Weak recommendation against an intervention	**Class III:** Evidence or general agreement that the given treatment or procedure is not useful/effective, and in some cases may be harmful	**Class 3: No benefit (moderate):** Benefit = Risk
Strong recommendation, against an intervention		**Class 3: Harm (strong):** Risk > Benefit

Adapted from Klugar et al.^11^ ACC, American College of Cardiology; AHA, American Heart Association; CCS, Canadian Cardiovascular Society; CHFS, Canadian Heart Failure Society; EO, expert opinion; ESC, European Society of Cardiology; GRADE, Grading of Recommendations, Assessment, Development and Evaluations; HFSA, Heart Failure Society of America; LD, limited data; RCT, randomized controlled trial.

## Classification and Staging of HF

Although the CCS HF guidelines last defined LVEF terminology in the 2017 update, all 3 guidelines have adopted LVEF categories consistent with the 2021 Universal Definition of HF: HFrEF (LVEF ≤ 40%); HFmrEF (41%-49%); HFpEF (≥ 50%); and HFimpEF (initial ≤ 40%, improved by at least 10% to ≥ 40%), with the exception of the ESC, which reserves the category of “HFimpEF” for HF with LVEF ≤ 40% improved to ≥ 50%.^15^ The CCS has not adopted or endorsed a staging system, whereas the AHA/ACC/HFSA uses HF stages A (at risk for HF), B (pre-HF), C (symptomatic HF), and D (advanced HF). This system is also endorsed by the ESC, but it is not incorporated into any recommendations and is only mentioned in the guideline supplement.

## Pharmacotherapy for HFrEF

### “Standard” HFrEF pharmacotherapy

**Figure 1:**
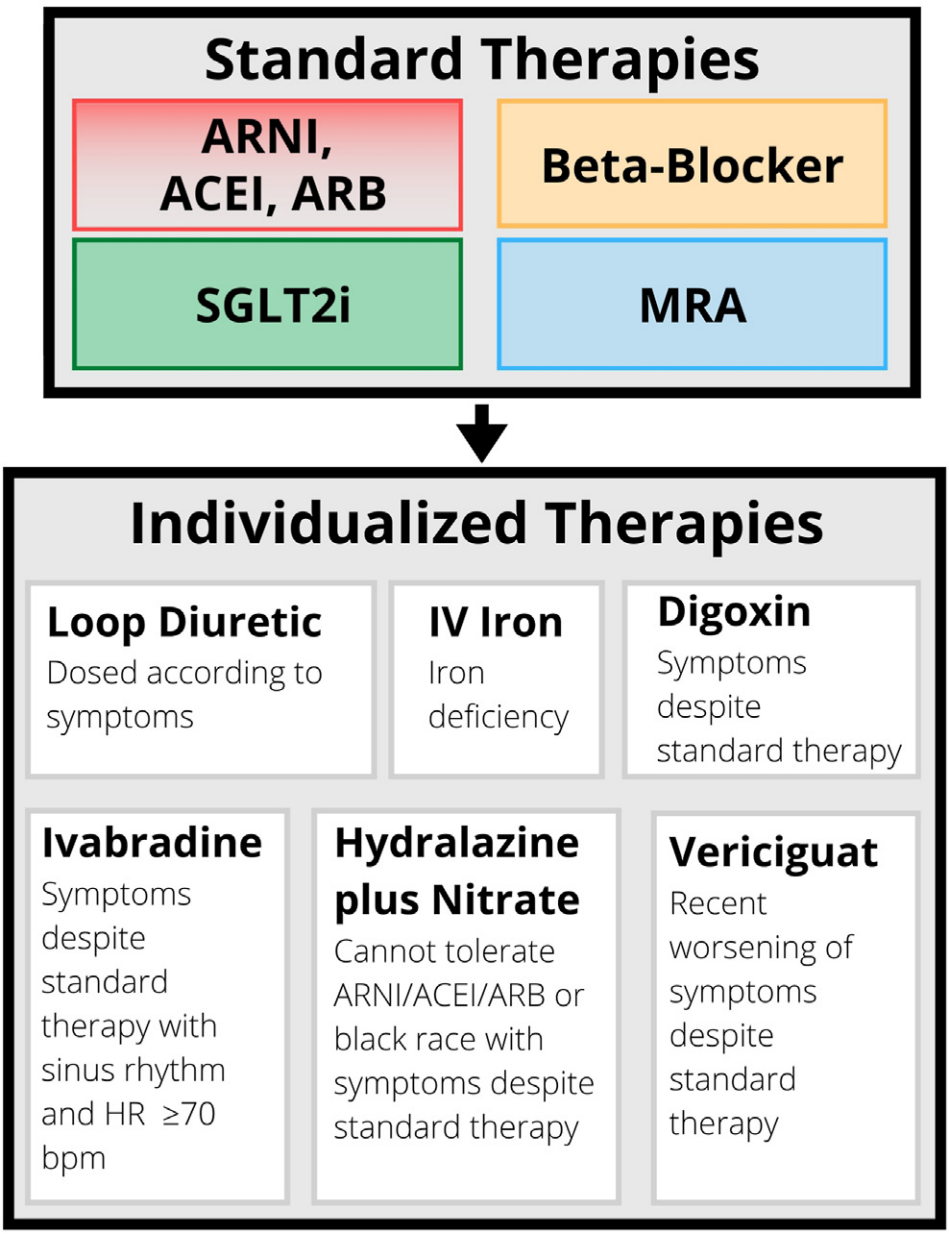
Consolidated international pharmacotherapy recommendations for heart failure with reduced ejection fraction. ACEI, angiotensin-converting enzyme inhibitor; ARB, angiotensin receptor blocker; ARNI, angiotensin receptor-neprilysin inhibitor; bpm beats per minute; HR, heart rate; IV, intravenous; MRA, mineralocorticoid receptor antagonist; SGLT2i, sodium/glucose cotransporter-2 inhibitor.

Management of patients with HFrEF is the area in which the 3 guidelines have the greatest consensus (Fig. 1). The guidelines are unanimous in recommending the combination of 4 standard therapy options for the treatment of HFrEF, as follows: angiotensin-converting enzyme inhibitor (ACEI), angiotensin receptor blocker (ARB), or angiotensin receptor-neprilysin inhibitors (ARNIs); beta-blocker; mineralocorticoid receptor antagonists (MRAs); and sodium-glucose cotransporter-2 inhibitors (SGLT2i’s). The CCS guidelines do, however, recommend that ACEI/ARB be switched to ARNIs prior to discharge in patients hospitalized with acute decompensated HF, and that ARNIs should be started in patients admitted with a new HFrEF diagnosis. The CCS guidelines also advise that starting with an ARNI rather than an ACEI/ARB may lead to more rapid optimization of therapy. Meanwhile, the ESC advises that ARNIs “may be considered as a first-line therapy instead of [ACEI],” without further specification of which conditions may warrant *de novo* initiation.

Both the CCS and the AHA/ACC/HFSA guidelines strongly recommend titrating standard therapies to target doses, whereas the ESC guidelines advise the same without providing formal recommendations on the best approach to achieve this end. All 3 guidelines provide tables of evidence-based drugs and doses, which have minor differences (Supplemental Table S1). Nebivolol is included as an evidence-based beta-blocker in the ESC guidelines, but not the CCS or AHA/ACC/HFSA guidelines.

Despite their consensus on what constitutes standard pharmacotherapy for HFrEF, all guidelines have stopped short of proposing a specific sequence to initiate and titrate medications. This reflects the lack of direct evidence to guide such recommendations, as each new class of medication historically has been compared with placebo in addition to the standard of care in the era when the trial was conducted. However, evidence and recognition are increasing that the benefits of HFrEF pharmacotherapy are additive to and independent of background medications, and that the pharmacologic effects of some medications may offset the adverse effects of others. For example, SGLT2i’s have similar efficacy irrespective of background medication use, including ARNIs and MRAs, and they can reduce the risk of hyperkalemia from renin-angiotensin-aldosterone system inhibitors.^16^^,^^17^ Various groups have proposed sequencing strategies based on these practical considerations.^18^^,^^19^ Given the large number of potential sequencing strategies (eg, ≥ 65 permutations, considering only order and number of medications to initiate on first encounter), comparison of every permutation in a randomized controlled trial (RCT) would be infeasible, and observational studies are hopelessly confounded for this purpose. In the absence of direct evidence from RCTs, modeling studies may inform guideline recommendations. The US Preventive Services Task Force recommendations for acetylsalicylic acid use in primary prevention of cardiovascular disease provide a recent example of guideline recommendations that have integrated best-available evidence from RCTs and modeling studies.^20^^,^^21^

A recent study by Shen et al. compared 13 potential sequences for standard HFrEF pharmacotherapy initiation, using a simple Markov model, based on data from 6 RCTs.^22^ All modeled sequences in this study involved fully titrating an individual medication before initiating the next medication in the sequence, and no sequence examined the simultaneous initiating of 3 or 4 medications at low doses. Notably, side effects were not included in the model (although the model partially accounted for treatment discontinuations by using the intention-to-treat results of RCTs). The absolute benefits of different strategies based on the modeling in the Shen et al. study are displayed in Table 3, along with projected Canadian population-level benefits. These population-level benefits are calculated based on the 2017 crude incidence of 106,500 new HF cases,^23^ a prevalence of HFrEF of ∼50% among all HF cases.^24^ As not all patients will want or be able to tolerate these medications, the results assuming both 100% (optimistic) and 75% (pessimistic) tolerability are displayed for illustrative purposes. The pessimistic scenario arbitrarily assumes 25% of the population received none of the HFrEF-sequence medications. Compared to no treatment and assuming 75% tolerability, at the population level, ∼3000 deaths are prevented in Canada each year by initiating a conventional sequence (ACEI → beta-blocker → MRA → ACEIΔARNI → SGLT2i) over 24 weeks, and a further ∼600 deaths per year would be averted by simply accelerating each step and altering the order of the sequence (MRA + beta-blocker → SGLT2i → ARNI over 10 weeks).

**Table 3:** Projected Canadian outcomes at 1 year in the Canadian population with different heart failure (HF) with reduced ejection fraction pharmacotherapy sequencing strategies

Strategy	HF hospitalization or CV death, incidence, %∗	Death		
		Incidence, %∗	Projected deaths with 100% adherence†	Projected deaths with 75% adherence†
No treatment	28.0	13.9	∼7600	∼7600
Traditional (ACEI → beta-blocker → MRA → ACEIΔARNI → SGLT2i; 24 wk)	12.9	6.5	∼3600 (–4000 vs no treatment)	∼4600 (–3000 vs no treatment)
Faster traditional (ACEI → beta-blocker → MRA → ACEIΔARNI → SGLT2i; 16 wk)	10.6	5.8	∼3100 (–500 vs traditional)	∼4200 (–400 vs traditional)
Direct ARNI (ARNI → beta-blocker → MRA → SGLT2i; 12 wk)	9.8	5.7	∼3000 (–600 vs traditional)	∼4100 (–500 vs traditional)
MRA- or SGLT2i-first (and incorporating direct ARNI into the sequence) (Various; 12 wk)	8.2 to 8.7	5.2 to 5.3	∼2800 (–800 vs traditional)	∼4000 (–600 vs traditional)
Starting with dual therapy (and incorporating direct ARNI into the sequence) (Various; 8–12 wk)	7.7 to 8.2	5.0 to 5.1	∼2700 (–900 vs traditional)	∼3900 (–700 vs traditional)

ACEI, angiotensin-converting enzyme inhibitor; ARNI, angiotensin receptor-neprilysin inhibitor; CV, cardiovascular; MRA, mineralocorticoid receptor antagonist; SGLT2i, sodium-glucose cotransporter-2 inhibitor.

∗ Estimates from the modeling study by Shen et al.^22^

† Projected Canadian population-level benefits calculated based on the 2017 crude incidence of 106,500 new HF cases in Canada, a prevalence of HF with reduced ejection fraction of ∼50% among all HF cases, and assuming 100% (optimistic) and 75% (pessimistic) adherence to medication regimens.

This modeling study offers several insights. First, the simple act of changing the order of medications without altering the final combination (“different journey, same destination”) could impact patient outcomes. Second, the conventional sequences implicitly mandated by insurance companies by requiring special authority and/or prior authorization do not represent those that provide the best efficacy outcomes, and such barriers may stall optimal implementation strategies. If the most-effective sequences cannot be implemented due to coverage considerations (eg, requiring an ACEI/ARB for 4 weeks prior to authorization for ARNI or SGLT2i coverage), the shorter titration schedules could still be implemented (ie, titrating over 16 weeks instead of 24 weeks). Third, at the individual level, all 8- to 12-week sequencing strategies modeled in this study produced comparable results (with the exception of the less-effective “direct ARNI” sequence [ARNI → beta-blocker → MRA → SGLT2i]), and decisions therefore should be informed by individual patient factors, a context in which the “spending function” framework can be useful for decision-making.^19^ In this framework, decisions for the next step in HFrEF pharmacotherapy optimization are based on the patient’s capacity to tolerate the medication, according to their blood pressure, heart rate, renal function, potassium concentration, out-of-pocket cost, and regimen complexity. For example, a patient with mild hyperkalemia would be best served by initiating an SGLT2i prior to starting an MRA or increasing the ARNI dose.^25^ Finally, starting 2 medications at once (rather than one) provides an approximately 0.5% to 1.5% further absolute reduction in the composite endpoint of cardiovascular death/HF hospitalizations, and a 0.1% to 0.5% further absolute reduction in death at 1 year. The tolerability of such an approach can be estimated based on the **S**afety, **T**ole**r**ability and Efficacy **o**f Up-Titratio**n** of **G**uideline-Directed Medical Therapies for Acute **H**eart **F**ailure (STRONG-HF) trial results.^26^ This trial randomized patients who were hospitalized for HF to local standard of care vs rapid initiation and up-titration of HFrEF pharmacotherapy, which consisted of initiating triple therapy at 50% target dose during HF admission, followed by up-titration of all 3 agents to target doses 2 weeks later. This intensive combination sequencing approach increased the risk of adverse events (mainly hypotension, hyperkalemia, and renal impairment) by an absolute 12% in the first 3 months, but without any increase in serious or fatal adverse events.

### “Individualized” HFrEF pharmacotherapy

In general, consensus has been reached regarding the use of other therapies for patients who remain symptomatic after optimization of standard therapies, coined as the term “individualized therapies” in the CCS guidelines (Fig. 1). The guidelines do not provide recommendations regarding optimal sequencing of these therapies, although they are implicitly given lower priority than standard therapies.

Unanimously weak recommendations are given for the use of digoxin in this population, reflecting the aging evidence, lack of mortality benefit, and risk of toxicity with this agent. Ivabradine is also recommended in patients with symptomatic HFrEF in sinus rhythm with a resting heart rate ≥ 70 beats per minute, with both the ESC and AHA/ACC/HFSA guidelines further specifying that LVEF should be ≤ 35%, which more strictly adheres to the inclusion criteria of the **S**ystolic **H**eart Failure Treatment with the **IF** Inhibitor Ivabradine **T**rial (SHIFT) trial. Notably, the Health Canada-approved indication for ivabradine is in patients with New York Heart Association (NYHA) class 2-3 HF with LVEF ≤ 35% and a resting heart rate ≥ 77 beats per minute, and many provincial drug plans reflect these criteria for coverage.^27^ Vericiguat is unanimously recommended for patients with “recently worsened” HF, although only the CCS also requires HF hospitalization within the past 6 months, reflecting the 84% of participants included based on this criterion in the landmark **V**er**ic**igua**t** Gl**o**bal Study in Subjects With Heart Failure With **R**educed Eject**i**on Fr**a**ction (VICTORIA) trial.^28^ The CCS’ recommendation is conditional upon formal approval of the agent for this indication in Canada (where it is also not yet available). The combination of hydralazine and isosorbide dinitrate is recommended by all guidelines in the case of intolerance of ACEI/ARNI/ARB, and in Black patients with “advanced” symptoms (NYHA class 3-4) despite optimized standard therapies. The ESC further specifies use in Black patients with NYHA class 3-4 symptoms and either LVEF < 45% with a dilated left ventricle, or LVEF ≤ 35%, strictly reflecting the **A**frican-American **He**art **F**ailure **T**rial (A-HeFT) trial inclusion criteria, from which these recommendations are derived.^29^ Finally, both the CCS and the AHA/ACC/HFSA guidelines weakly recommend omega-3 fatty acids in patients with HFrEF, based on the **G**ruppo **I**taliano per lo **S**tudio della **S**opravvivenzanell’**I**nsufficienza cardiaca-**H**eart **F**ailure (GISSI-HF) trial, with the CCS guidelines further noting the high variability in omega-3 content of over-the-counter products.^30^ None of the guidelines have made recommendations for (or against) omecamtiv mecarbil, despite availability of the results of the **G**lobal **A**pproach to **L**owering **A**dverse **C**ardiac Outcomes **T**hrough **I**mproving **C**ontractility in **H**eart **F**ailure (GALACTIC-HF) trial.^31^ Both the CCS and ESC did note modest benefit and uncertainty regarding its role, whereas AHA/ACC/HFSA highlighted its use in advanced HF as an area of future research.

## Continuation of HFrEF Pharmacotherapy in HFimpEF

All guidelines acknowledge HFimpEF as a distinct category, consistent with the Universal Definition of HF.^15^ Based on the results of the A Pilo**t** Feasibility Study in **Re**covere**d H**eart **F**ailure (TRED-HF) trial, the ESC guidelines advise (without formal recommendations) and the AHA/ACC/HFSA guidelines recommend that patients with HFimpEF should continue to receive their HFrEF medications indefinitely.^32^ TRED-HF was a pilot, open-label RCT of 51 patients with previous HFrEF secondary to dilated cardiomyopathy and current HFimpEF (defined as NYHA class 1; LVEF ≥ 50%; N-terminal prohormone of brain natriuretic peptide [NT-proBNP] < 250 ng/L) receiving at least one HFrEF medication. Patients were randomized to sequential discontinuation of HFrEF medications over 4 months vs continuation of all current HFrEF medications. Over the 6-month follow-up, 44% of patients in the discontinuation group, vs 0% in the continuation group, had clinically significant relapse (based on changes in HF signs or symptoms, changes in left ventricular dimension or function, or elevated natriuretic peptides). A subgroup analysis of patients with HFimpEF in the **D**apagliflozin in H**e**art Fai**l**ure w**i**th Preser**ve**d and Mildly **R**educed Ejection Fraction (DELIVER) trial demonstrated a statistically significant reduction in the primary outcome of cardiovascular death or worsening HF with dapagliflozin, compared to placebo, consistent with its effect across the LVEF spectrum and further affirming the efficacy of medications used for HFrEF in the HFimpEF population.^33^

Prior to the availability of the TRED-HF or DELIVER trial results, the CCS 2017 guidelines described (without formal recommendations) select populations in which consideration could be given to withdraw HF medications after 6-12 months of therapy with normalization of LVEF and left ventricular dimensions in asymptomatic (NYHA class 1) patients. Although neither the TRED-HF nor DELIVER trial results directly contradict this guidance, they do suggest that caution is warranted with this approach. This situation highlights an issue common to all guidelines, which is that their recommendations and discussions often become outdated as new evidence emerges. Given that clinicians who rely on these guidelines may not evaluate emerging evidence or keep up with guidelines from other countries, this may result in suboptimal care in the period between guideline revisions and their dissemination. Consequently, guideline-development processes could be improved by implementing systems to update their content upon the publication of major evidence. At a minimum, guidelines could update such recommendations with a disclaimer regarding the presence of new evidence (with a link to said evidence or to newer guidelines incorporating this evidence) in these cases. The World Health Organization living guidelines for the management of COVID-19 provide an ambitious example of GRADE-based guidelines that have been updated continuously to incorporate rapidly evolving evidence.^34^ Regardless of the exact methodology implemented, further efforts are required to improve the timeliness of guideline recommendations and updates.

## Pharmacotherapy for HFpEF

**Figure 2:**
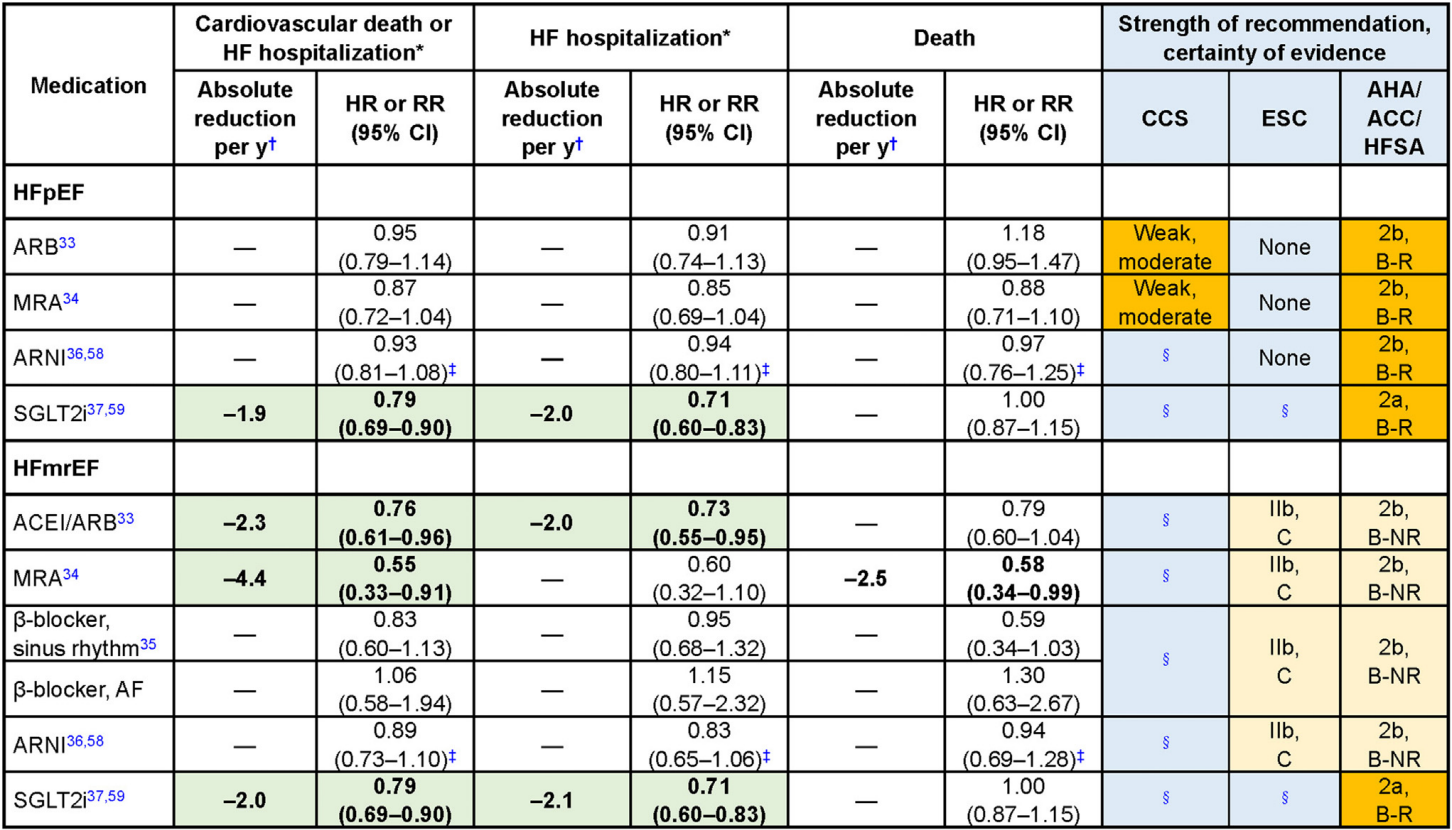
Heart failure (HF) with preserved ejection fraction (HFpEF) guideline and HF with mildly reduced EF (HFmrEF) recommendations and annual absolute difference by medication. ∗Cardiovascular hospitalization, rather than HF hospitalization, in the case of β-blockers. ^†^Approximate standardized annual absolute risk reduction calculated applying statistically significant relative risk reductions to incidence rate in Candesartan in Heart Failure Assessment of Reduction in Mortality and Morbidity (CHARM-Preserved)^36^ placebo subgroups with left ventricular ejection fraction ≥ 50% and 41%-49%. ^‡^Analysis from Prospective Comparison of ARNI with ARB Global Outcomes in Heart Failure With Preserved Ejection Fraction (PARAGON-HF) subgroups of patients with ejection fraction > 52.5%-62.5% and > 62.5% (pooled for HFpEF) and ≥ 42.5% for HFmrEF. Hazard for ratio for death is cardiovascular death (subgroup data for all-cause death not available). ^§^Guideline published before evidence available. ACC, American College of Cardiology; ACEI, angiotensin-converting enzyme inhibitor; AHA, American Heart Association; ARB, angiotensin receptor blocker; ARNI, angiotensin receptor-neprilysin inhibitor; B-NR, level B nonrandomized; B-R, level B randomized; CCS, Canadian Cardiovascular Society; CHFS, Canadian Heart Failure Society; CI, confidence interval; ESC, European Society of Cardiology; HFSA, Heart Failure Society of America; HR, hazard ratio; MRA, mineralocorticoid receptor antagonist; RR, risk ratio; SGLT2i, sodium-glucose transport protein 2 inhibitor.

The term “HFpEF” was first used in the **C**andesartan in **H**eart Failure **A**ssessment of **R**eduction in **M**ortality and Morbidity (CHARM-Preserved) trial to evaluate pharmacotherapy in patients “without reduced LVEF,” defined as HF and an LVEF > 40%.^35^ Subsequent trials in “HFpEF” have used various thresholds (> 40%, ≥ 45%, and ≥ 50%) to define “preserved” LVEF.^35^ The current universal definition of HFpEF employed by all 3 guidelines requires an LVEF ≥ 50%. Despite this requirement, substantial discordance is currently present in the recommendations for HFpEF pharmacotherapy across guidelines. Figure 2 provides a comparison of recommendations and estimates of effect based on best-available evidence.^39^, ^40^, ^43^, ^44^, ^45^, ^46^, ^47^ All 3 guidelines unanimously recommend blood pressure control in patients with HFpEF, given that hypertension is the most common etiology for development and progression of HFpEF. Both the CCS and AHA/ACC/HFSA defer to hypertension guidelines for recommendations on blood pressure targets, whereas the ESC does not offer a target recommendation, owing to lack of evidence in patients with concomitant HF.

Both the CCS 2017 and the AHA/ACC/HFSA guidelines give weak recommendations for an ARB (based on the CHARM-Preserved trial) and/or an MRA (based on the **T**reatment **o**f **P**reserved **Ca**rdiac Function Heart Failure With an Aldos**t**erone Antagonist [TOPCAT] trial) in patients with HFpEF.^43^ The AHA/ACC/HFSA guidelines specifically note the greater likelihood of benefit in patients on the “lower end of the LVEF spectrum” (ie, near or below 50%). The CCS guidelines also acknowledge the neutral results found with irbesartan in the **I**rbesartan in Heart Failure with **Preserve**d Ejection Fraction Study (I-PRESERVE) trial.^44^ Given that the primary outcomes in these trials were neutral, the ESC states that their findings are hypothesis-generating only, and consequently, they do not provide any recommendations for these medications in HFpEF. Conversely, the CCS and the AHA/ACC/HFSA emphasize a statistically significant reduction in the secondary outcome of HF hospitalization in favour of treatment in the CHARM-Preserved and TOPCAT trials. The CCS and AHA/ACC/HFSA guidelines also note a statistically significant benefit in the primary composite outcome within the Americas subgroup of the TOPCAT trial in the context of likely enrollment of patients without (or with less-severe) HF, and other protocol violations, uncovered in the Russia/Georgia sites.^45^^,^^46^

Based on the inconclusive results of the **P**rospective Comparison of **AR**NI with **A**RB **G**lobal **O**utcomes in Heart Failure With Preserved Ejectio**n** Fraction (PARAGON-HF) trial comparing use of ARNIs to use of ARBs in patients with HF and LVEF ≥ 45%, the CCS and ESC guidelines did not make any formal recommendation for use of ARNIs in HFpEF, awaiting further analyses or trials for more conclusive findings.^39^ Conversely, the AHA/ACC/HFSA guidelines provide a weak recommendation for ARNI use in this population, citing a “signal of benefit” for the secondary outcome of HF hospitalization (rate ratio 0.85, 95% confidence interval [CI] 0.72-1.00, *P* = 0.056), along with subgroup differences indicating benefit in women or patients with an LVEF of 45%-57%.

Results of the **Emp**agliflozin **O**utcome T**r**ial in Patients with Chr**o**nic Hea**r**t Failure with **Preserved** Ejection Fraction (EMPEROR-Preserved) trial, which included patients with HF and LVEF > 40% (ie, either HFpEF or HFmrEF), were presented and published on the same day as the ESC guidelines (and subsequent to publication of the CCS 2021 guidelines). Given this timing, only the AHA/ACC/HFSA provide recommendations for SGLT2i’s for HFpEF.^40^ Given that the primary composite outcome was positive in this trial and in the DELIVER trial of dapagliflozin in patients with HF and LVEF > 40%, consensus across all 3 guidelines may be reached in the future on recommendations for this class of medications in HFpEF.^47^ As with HFrEF, none of the guidelines provide specific recommendations for pharmacotherapy sequencing in HFpEF.

The overarching differences in recommendations for HFpEF between guidelines reflect the lowlevel of certainty of evidence for medications in this condition. Clinicians may be left confused when guideline recommendations differ and in cases in which multiple medications have weak recommendations, without clear guidance on their relative or cumulative benefits. The utility of “weak/conditional recommendations” could be improved by embracing the role of shared decision-making. To that end, every weak recommendation should be accompanied by sufficient information to facilitate shared decision-making with patients, ideally in the form of a decision aid. In cases in which no decision aids exist (as with HFpEF/HFmrEF pharmacotherapy at the time of this writing), guidelines should describe the uncertainty of the evidence and provide a synopsis of known benefits, harms, and other information needed to make a shared decision. So, rather than patients with the same condition being treated differently based on which guideline their clinicians follow, they could receive treatment that is individualized according to their values and preferences, no matter where they reside.

## Pharmacotherapy for HFmrEF

CCS guidelines have not yet broached specific recommendations for HFmrEF pharmacotherapy, although the 2017 guidelines highlighted this as an area for future research. In general, evidence for managing HFmrEF is derived from subgroup analyses from landmark trials of “HFpEF” (many of which included patients based on their having an LVEF > 40% or ≥ 45%). Recognizing this limited evidence, the ESC and AHA/ACC/HFSA guidelines weakly recommended ACEI/ARBs, ARNIs, MRAs, and beta-blockers in patients with HFmrEF (Figure 2). As previously noted, only the AHA/ACC/HFSA guideline was published after the EMPEROR-Preserved trial results were published, so this is the only guideline to provide a recommendation for SGLT2i use in HFmrEF. As in HFrEF and HFpEF, none of the guidelines provides specific recommendations for sequence of initiation of pharmacotherapy in this population.

The HFmrEF recommendations highlight a key issue with the methodology used to rate quality of evidence by both the AHA/ACC/HFSA and ESC guidelines. The AHA/ACC/HFSA recommendations rate the level/certainty of evidence for SGLT2i and the other medications (ACEI/ARB, ARNI, MRA, and beta-blocker) as “moderate quality” (level B, randomized [B-R] and level B-nonrandomized [B-NR], respectively). However, the SGLT2i recommendation is based on the high-quality EMPEROR-Preserved trial (now further supported by the DELIVER trial), whereas the evidence for the other medications is an amalgamation of post hoc subgroup analyses and secondary analyses from trials with initially neutral primary outcome results. As noted, the ESC provided similar HFmrEF pharmacotherapy recommendations, but without providing a clear explanation for their difference in approach compared to the skepticism shown toward HFpEF evidence. As discussed below, the incorporation of use of the GRADE framework would enhance transparency and help clarify the reasoning behind such recommendations.

These idiosyncrasies could be better understood by universally incorporating use of the GRADE framework, which would facilitate transparent reporting of recommendation rationale, as well as reasons to downgrade the rating of level of certainty of evidence.^48^ This framework offers a systematic approach to the development of guideline recommendations, including a transparent assessment of the certainty of evidence for individual recommendations. Through the adoption of this framework, guidelines can better describe the rationale and underlying evidence informing each recommendation. Although the approaches currently employed by the ESC and AHA/ACC/HFSA guidelines appear simpler and more objective, the GRADE framework allows for explicit and transparent consideration of limitations in the body of evidence (risk of bias, inconsistency, imprecision, indirectness, and publication bias) that warrant downgrading of the level of certainty of evidence. Although this decision requires subjective judgement, panelists can achieve high reproducibility after minimal training.^49^ Ultimately, adoption of the GRADE framework will contribute to guidelines that are more systematic, transparent, and reflective of the available evidence and local contexts that inform recommendations.

## Special Considerations

### Identification and treatment of iron deficiency in HF

Iron deficiency is associated with worse quality of life, survival, and risk of hospitalizations among patients with HF, irrespective of their anemia status.^50^ The ESC guideline recommends periodic screening for anemia and iron deficiency; the AHA/ACC/HFSA notes that anemia should be a routine part of baseline assessment; and the CCS notes to only investigate and treat reversible causes of anemia. The guidelines are unanimous in recommending against erythropoiesis-stimulating agents in patients with HF and anemia, except possibly in those with other non-HF indications. The guidelines are unanimous that intravenous iron should be used in patients with HFrEF and iron deficiency (defined as ferritin < 100 ng/mL, or transferrin saturation < 20%, with ferritin 100-299 ng/mL), irrespective of anemia status. The ESC guidelines more specifically recommend the use of intravenous ferric carboxymaltose in patients with iron deficiency and (i) symptomatic chronic HF with LVEF ≤ 45% (criterion 1), (ii) patients with HF, LVEF < 50%, and a recent HF hospitalization (criterion 2), or (iii) during HF hospitalization and continued postdischarge (criterion 3).

The evidence cited for these recommendations includes a meta-analysis of patients with iron deficiency and HF with LVEF < 45% for criterion 1,^51^ and the **A** Randomized, Double-blind Placebo-controlled Trial Comparing the E**ff**ect of **I**ntravenous **F**erric Carboxy**m**altose on Hospitalizations and Mortality in Iron-deficient Subjects Admitted for **A**cute **H**eart **F**ailure (AFFIRM-AHF) trial^52^ for criteria 2 and 3, although how the AFFIRM-AHF trial justifies the use of intravenous iron in patients with LVEF > 50% in unclear, as these patients were excluded from the trial. The recommendation to use ferric carboxymaltose is presumably based on the use of this specific agent in the largest intravenous iron trials available at the time of publication. However, no evidence shows that one formulation of intravenous iron is better than any other, and issues with cost and access often preclude preferential use of one formulation. This lack of evidence is corroborated by the later-published results of the Effectiveness of **I**nt**r**avenous Ir**on** Treat**m**ent Versus Standard Care in P**a**tie**n**ts with Heart Failure and Iron Deficiency (IRONMAN) trial. This study, which was larger than the aforementioned ferric carboxymaltose trials, demonstrated the efficacy of another iron formulation (ferric derisomaltose) for this indication.^53^

Despite this broad consensus concerning intravenous iron use, little emphasis is placed on this intervention, despite the fact that it provides greater quality-of-life improvements than any other pharmacologic intervention in patients with HFrEF.^54^ For example, the 2021 CCS and 2022 AHA/ACC/HFSA guidelines have separate decision trees with “individualized therapies” that feature other medications (eg, digoxin, vericiguat), but not intravenous iron (which was only described in the AHA/ACC/HFSA separate comorbidity treatment algorithm). Only the ESC includes intravenous iron in their HFrEF treatment algorithm, although this mention is relegated to a later section on managing noncardiovascular comorbidities, which could be overlooked, given the length of the document.

The importance of such quality-of-life improvements is demonstrated in a scoping review that examined the values and preferences of patients with HFrEF^10^—with patients in one study ranking quality of life as one of the most important treatment attributes (ranked 4th out of 18 attributes), and another study indicating that 92% of patients were willing to accept some increased risk of dying in exchange for improved quality of life (although this proportion varied with the magnitude of these tradeoffs and the cost of the medication). These quality-of-life data are also reflective of the growing body of literature examining patient values and preferences regarding HFrEF pharmacotherapy, which can be used to inform future guideline iterations regarding the prioritization of interventions.^59^, ^9^, ^11^, ^41^

### Sodium restriction

Guidelines are unanimous in advising sodium restriction, but they differ in their target range or limit. The 2017 CCS guidelines make a weak recommendation to restrict dietary sodium intake to 2-3 g/d in patients with HF. The ESC guidelines did not make a formal recommendation, but suggested avoiding excess sodium intake (> 2 g/d). The AHA/ACC/HFSA guidelines make a moderate-strength recommendation that “excess sodium” be avoided, with no particular limit suggested. However, separate from the HF guidelines, on their website, the AHA does advise most adults to consume ≤ 2.3 g/d of sodium, with an “ideal limit” of ≤1.4 g/d.^59^ All 3 guidelines comment on the high level of uncertainty regarding the benefits and harms of sodium restriction, owing to inadequate evidence.

The **S**tudy **O**f **D**ietary **I**ntervention **U**nder 100 **m**mol in **H**eart **F**ailure (SODIUM-HF) trial, published in 2022, may guide future sodium restriction recommendations.^9^ This trial randomized 841 patients with HF across the LVEF spectrum to a target dietary sodium intake < 1.5 g/d (implemented by providing country-specific meal plans and menus) or usual care (general advice to restrict dietary sodium). At 1 year, the mean achieved daily sodium intake was approximately 1.6 g vs 2 g with intervention and control, respectively. No significant difference occurred in the primary outcome of death or cardiovascular hospitalization/emergency department visit or any of the components. Although the stricter target group had a statistically significant improvement in quality of life, this result should be taken “with a grain of salt,” given that this was an open-label trial with differential follow-up. Overall, these findings are consistent with existing guideline recommendations (none of which recommended restrictions of < 1.5 g/d), although they do conflict with the “ideal limit” of ≤ 1.4 g/d proposed by the AHA. Moreover, the daily sodium intake achieved in the control group closely reflects the range recommended by the CCS guidelines.

Whether this trial alone will lead to revised recommendations in future guideline iterations remains to be seen. In contrast to the uncertainty regarding HFmrEF and HFpEF pharmacotherapy, which pertains to imprecision around the effect estimate, the uncertainty reflected by different sodium-restriction targets is related to the direction of effect. The fact that the ESC and AHA/ACC/HFSA are hesitant to make formal recommendations under such circumstances is not surprising. Nonetheless, a reasonable proposal may be for a weak recommendation that patients aim for a range of sodium intake of ∼1.4-3 g/d (based on the range achieved in the SODIUM-HF trial). The use of a range (an approach already adopted by the CCS) reflects the uncertain state of evidence and could also harmonize current guideline recommendations. Additionally, irrespective of consensus on a specific range, guidelines could still make strong recommendations in favour of shared decision-making in such instances. For example, a strong recommendation could be made in favour of having discussion with patients regarding the fact that the benefits of sodium restriction targets are uncertain, but that stricter restrictions (eg, 1.5 g/d) may reduce symptoms, hospitalizations, and loop diuretic requirements, compared to more-liberal restrictions (eg, > 4 g/d). Also part of the discussion should be the fact that some patients find that stricter restrictions result in a diet that is less enjoyable, more time-consuming, or more expensive (which can all impair quality of life).

## Summary

This comparison of 3 major international HF guidelines demonstrated broad agreement among the 3, but it also identified several key areas for improvement. First, the unanimous uncertainty surrounding optimal HFrEF treatment sequencing could be informed by model-based estimates in the absence of RCT data. Second, to ensure that guidelines are transparent and up-to-date, adoption of use of the GRADE framework, along with mechanisms to rapidly update recommendations based on emerging evidence, is needed. Third, guidelines could facilitate shared decision-making by providing syntheses of the benefits and harms of treatments that informed the recommendations. Fourth, the relative importance of different therapies could be more accurately identified via further considerations of patient values and preferences. Finally, to improve the quality of recommendations when the level of certainty of evidence is low, guidelines could make nuanced weak recommendations and transparently disclose such uncertainties. Additionally, the review provided therapeutic guidance to clinicians navigating the complexities of HF management. Adoption of these recommendations for guideline developers could further assist clinicians in providing evidence-based collaborative care for patients with HF.
