# Supplementary material for: Heart Failure Management in 2023: A Pharmacotherapy- and Lifestyle-Focused Comparison of Current International Guidelines
Source: CJC Open. 2023 May 26;5(8):629–40. doi: 10.1016/j.cjco.2023.05.008 (PMC10502425; doi:10.1016/j.cjco.2023.05.008)
Supplement: Supplementary Material [file mmc1.pdf]

Supplemental Table S1: Dosing Recommendations for Heart Failure with Reduced Ejection Fraction Medications

Class	Medication	CCS 2021		ACC		ESC	
		Start	Target	Start	Target	Start	Target
**Angiotensin-converting enzyme inhibitors (ACEI)**	**Captopril**	-	-	6.25 mg three times daily	50 mg three times daily	6.25 mg three times daily	50 mg three times daily
	**Enalapril**	1.25-2.5 mg twice daily	10 mg twice daily/20 mg twice daily (New York Heart Association Class IV)	2.5 mg twice daily	10–20 mg twice daily	2.5 mg twice daily	10-20 mg twice daily
	**Fosinopril**	-	-	5–10 mg once daily	40 mg once daily	-	-
	**Lisinopril***	2.5-5 mg once daily	20-35 mg daily	2.5–5 mg once daily	20–40 mg once daily	2.5-5 mg once daily	20-35 mg once daily
	**Perindopril**	2-4 mg once daily	4-8 mg once daily	2 mg once daily	8–16 mg once daily	-	-
	**Quinapril**	-	-	5 mg twice daily	20 mg twice daily	-	-

	**Ramipril**	1.25-2.5 mg twice daily	5 mg twice daily	1.25–2.5 mg once daily	10 mg once daily	2.5 mg twice daily	5 mg twice daily
	**Trandolapril**	1-2 mg once daily	4 mg once daily	1 mg once daily	4 mg once daily	0.5 mg once daily	4 mg once daily
**Angiotensin receptor blockers (ARB)**	**Candesartan**	4-8 mg once daily	32 mg once daily	4–8 mg once daily	32 mg once daily	4 mg once daily	32 mg once daily
	**Losartan**	-	-	25–50 mg once daily	50–150 mg once daily	50 mg once daily	150 mg once daily
	**Valsartan**	40 mg twice daily	160 mg twice daily	20–40 mg once daily	160 mg twice daily	40 mg twice daily	160 mg twice daily
**Angiotensin-converting enzyme inhibitors (ARNI)**	**Sacubitril-valsartan**	50-100 mg twice daily (dose round ed)	200 mg twice daily (dose rounded)	49 mg sacubitril and 51 mg valsartan twice daily (therapy may be initiated at 24 mg sacubitril and 26 mg	97 mg sacubitril and 103 mg valsartan twice daily	49/51 mg twice daily (Sacubitril/valsartan may have an optional lower starting dose of 24/26 mg twice daily for those with a history of	97/103 mg twice daily

				valsartan twice daily)		symptomatic hypotension)	
**Beta-blockers**	**Carvedilol**	3.125 mg twice daily	25 mg twice daily/50 mg twice daily (> 85 kg)	3.125 mg twice daily	25–50 mg twice daily	3.125 mg twice daily	25 mg twice daily (a maximum dose of 50 mg twice daily can be administered to patients weighing over 85 kg)
	**Carvedilol CR**	-	-	10 mg once daily	80 mg once daily	-	-
	**Bisoprolol**	1.25 mg once daily	10 mg once daily	1.25 mg once daily	10 mg once daily	1.25 mg once daily	10 mg once daily
	**Metoprolol (CR/XL)**	12.2-25 mg once daily	200 mg once daily	12.5–25 mg once daily	200 mg once daily	12.5-25 mg once daily	200 mg once daily
	**Nebivolol****	-	-	-	-	1.25 mg once daily	10 mg once daily
**Mineralocorticoid receptor**	**Spironolactone**	12.5 mg	25-50 mg once daily	12.5–25 mg once daily	25–50 mg once daily	25 mg once daily (Spironolactone	50 mg once daily

**antagonists (MRAs)**		once daily				has an optional starting dose of 12.5 mg in patients where renal status or hyperkalaemia warrant caution)	
	**Eplerenone**	25 mg once daily	50 mg once daily	25 mg once daily	50 mg once daily	25 mg once daily	50 mg once daily
**Sodium-glucose Cotransporter 2 Inhibitors (SGLT2i)**	**Dapagliflozin**	10 mg once daily	10 mg once daily	10 mg once daily	10 mg once daily	10 mg once daily	10 mg once daily
	**Empagliflozin**	10 mg once daily	10-25 mg once daily	10 mg once daily	10 mg once daily	10 mg once daily	10 mg once daily
	**Canagliflozin**	100 mg once daily	100-300 mg once daily	-	-	-	-
**Hyperpolarization-activated cyclic nucleotide-gated channel blockers**	**Ivabradine**	2.5-5 mg twice daily	7.5 mg twice daily	5 mg twice daily	7.5 mg twice daily	5 mg twice daily	7.5 mg twice daily
**Vasodilator-Nitrates**	**Hydralazine and isosorbide dinitrate**	10-37.5 mg	75-100 mg TID or	20–30 mg isosorbide dinitrate and	120 mg isosorbide dinitrate total	37.5 mg three times daily/20	75 mg three times daily/40 mg

		TID/10-20 mg TID	QID/40 mg TID	25–50 mg hydralazine 3–4 times daily	daily in divided doses and 300 mg hydralazine total daily in divided doses	mg three times daily	three times daily
**Glycosides**	**Digoxin**	0.0625-0.125 mg once daily	Not applicable: monitor for toxicity	0.125–0.25 mg daily (modified according to monogram)	Individualized variable dose to achieve serum digoxin concentration 0.5–<0.9 ng/mL	62.5 mg once daily	250 mg once daily
**Soluble guanylate cyclase activator**	**Vericiguat**	2.5 mg once daily	10 mg once daily	2.5 mg once daily	10 mg once daily	2.5 mg once daily	10 mg once daily

*ESC: A higher dose has been shown to reduce morbidity/mortality compared with a lower dose of the same drug, but there is no substantive randomized, placebo-controlled trial and the optimum dose is uncertain
**ESC: Indicates a treatment not shown to reduce CV or all-cause mortality in patients with heart failure (or shown to be non-inferior to a treatment that does).
